# Preventive effect of ecabet sodium on low-dose aspirin-induced small intestinal mucosal injury: a randomized, double-blind, pilot study

**DOI:** 10.1186/s12876-018-0923-7

**Published:** 2019-01-08

**Authors:** Kazuhiro Ota, Toshihisa Takeuchi, Yuichi Kojima, Satoshi Harada, Yuki Hirata, Noriaki Sugawara, Sadaharu Nouda, Kazuki Kakimoto, Takanori Kuramoto, Kazuhide Higuchi

**Affiliations:** Second Department of Internal Medicine, Osaka Medical College, 2-7 Daigaku-Machi, Takatsuki, Osaka, 569-8686 Japan

**Keywords:** Aspirin, Ecabet sodium, Capsule endoscopy, Small intestinal mucosal injury, Small intestine

## Abstract

**Background:**

We aimed to investigate how high-dose ecabet sodium affects low-dose aspirin-induced small intestinal mucosal injury in healthy volunteers.

**Methods:**

Healthy volunteers were enrolled randomly into one of two groups with the following drug regimens for 2 weeks: group A, low-dose aspirin once per day and group B, low-dose aspirin and 4.0 g of ecabet sodium. Small bowel capsule endoscopy was performed before and 2 weeks after low-dose aspirin administration.

**Results:**

A significant difference was found in the median number [range] of small intestinal lesions between baseline and two weeks after low-dose aspirin administration in group A (baseline: 1 [0–5], after: 5 [1–11]; *p* = 0.0059) but not in group B (baseline: 0.5 [0–9], after: 3 [0–23]; *p* = 0.0586). In group B, although the median number [range] of lesions in the first tertile of the small intestine did not increase two weeks after low-dose aspirin administration (baseline: 0 [0–4], after: 1.5 [0–8]; *p* = 0.2969), the number of lesions in the second and third tertiles of the small intestine increased significantly (baseline: 0 [0–5], after: 2 [0–15]; *p* = 0.0469).

**Conclusions:**

Ecabet sodium had a preventive effect on low-dose aspirin-induced small intestinal mucosal injury in the upper part of the small intestine.

**Trial registration:**

ISRCTN 99322160, 01/10/2018.

## Background

Low-dose aspirin (LDA) exerts antiplatelet effects by inhibiting cyclooxygenase-1 activity and suppressing prostaglandin production; however, the reduction in prostaglandins causes whole intestinal mucosal injury [1–3]. It is recommended that LDA users concurrently use a proton pump inhibitor (PPI) to prevent LDA-induced upper gastrointestinal mucosal injury [4, 5]. The inhibition of gastric acid secretion cannot prevent small intestinal mucosal injury that is not associated with gastric acid, and a strategy to inhibit LDA-induced small intestinal mucosal injury has not been established. Recently, it has been reported that several gastric mucoprotective drugs are effective for preventing LDA-induced small intestinal mucosal injury [5–10]. Rebamipide, a gastric mucoprotective drug, effectively prevents LDA-induced gastrointestinal mucosal injury by stimulating production of prostaglandin and epidermal growth factor [11]. We reported that rebamipide inhibited increases in fecal levels of calprotectin, an inflammatory biomarker of the lower intestinal mucosa for LDA [6, 12].

Ecabet sodium (ES), a gastric mucoprotective drug that acts locally as an antiulcer agent, has anti-pepsin activity. ES binds to proteins to form a complex that is resistant to the peptic activity of gastric juice, increasing the capacity of the gastric mucosa to synthesize prostaglandin E2 and/or prostacyclin to enhance gastric mucosal defensive factors, including mucosal blood flow, mucosal adherent mucus, and mucosal bicarbonate secretion [13]. As these mechanisms share some features with those of rebamipide, we hypothesized that ES might also prevent small intestinal mucosal injury.

Although we previously reported that ES (100 mg/kg) did not inhibit indomethacin-induced small intestinal mucosal injury in rats [14], there are no such reports in humans. We suspected that the regular dose of ES might be too low to be effective in the human small intestine. Therefore, the aim of this preliminary clinical study in healthy volunteers was to investigate how high-dose ES affected LDA-induced small intestinal mucosal injury.

## Methods

### Subjects

Concerning the inclusion criteria of healthy adults were aged between 20 and 65 years at the time of providing consent, freely provided informed consent on the basis of their full understanding of the study protocol, and had no history of medication use during the month before enrolment. We excluded persons with history of peptic ulcer or gastrointestinal bleeding, significant hepatic, renal, heart, or respiratory disease, history of gastrointestinal surgery other than appendectomy, use of oral histamine H2-receptor antagonists, gastrointestinal kinetic agents, or gastric mucoprotective drugs within 2 weeks prior to the study, use of oral non-steroidal anti-inflammatory drugs (NSAIDs), steroids, anticholinergic drugs, anticancer drugs, or antithrombotic drugs within 4 weeks prior to the study, alcohol or chemical dependency, history of gastrointestinal obstruction, refusal to consent to the surgery that would be required if the capsule endoscope was retained in the body, and determination by the investigator, at his/her discretion, that a subject was ineligible for participation in the study for any reason. All criteria were met by 30 subjects. All subjects received oral and written information about the study and signed informed consent forms. This study was conducted prospectively at Osaka Medical College Hospital between October 2011 and February 2012. The study was performed in accordance with the 1975 Declaration of Helsinki (as revised in 1983) and CONSORT guidelines, and the protocol was approved by the ethics review committee at Osaka Medical College (No. 730) and was registered in the University Hospital Medical Information Network Clinical Trial Registry (UMIN000033984).

### Study design

This was a prospective randomized trial investigating the preventive effect of ES for LDA-induced small intestinal mucosal injury. The 30 subjects were divided into two groups (A and B; *n* = 15 each) and were instructed to take the study drug(s) for 2 weeks. The subjects in group A received LDA (100 mg) once daily, and those in group B received LDA and 4.0 g of ES (1.0 g four times daily; GASTROM® Granules 66.7%, Mitsubishi Tanabe Pharma Corporation, Osaka, Japan). The dosage of aspirin was determined on the basis of the dosage recommended for antithrombotic activity in cardiovascular and cerebrovascular diseases [15–17]. The dosage of ES, which was double the regular dose, was determined on the basis of the following: 1) a regular dose of rebamipide could not heal LDA-induced small intestinal mucosal injury [8]; and 2) ES is not absorbed from the intestine and is excreted in the feces [18].

Small bowel capsule endoscopy (SBCE) was performed before and 2 weeks after drug administration.

### Evaluation of small intestinal mucosal lesions

**Fig. 1:**
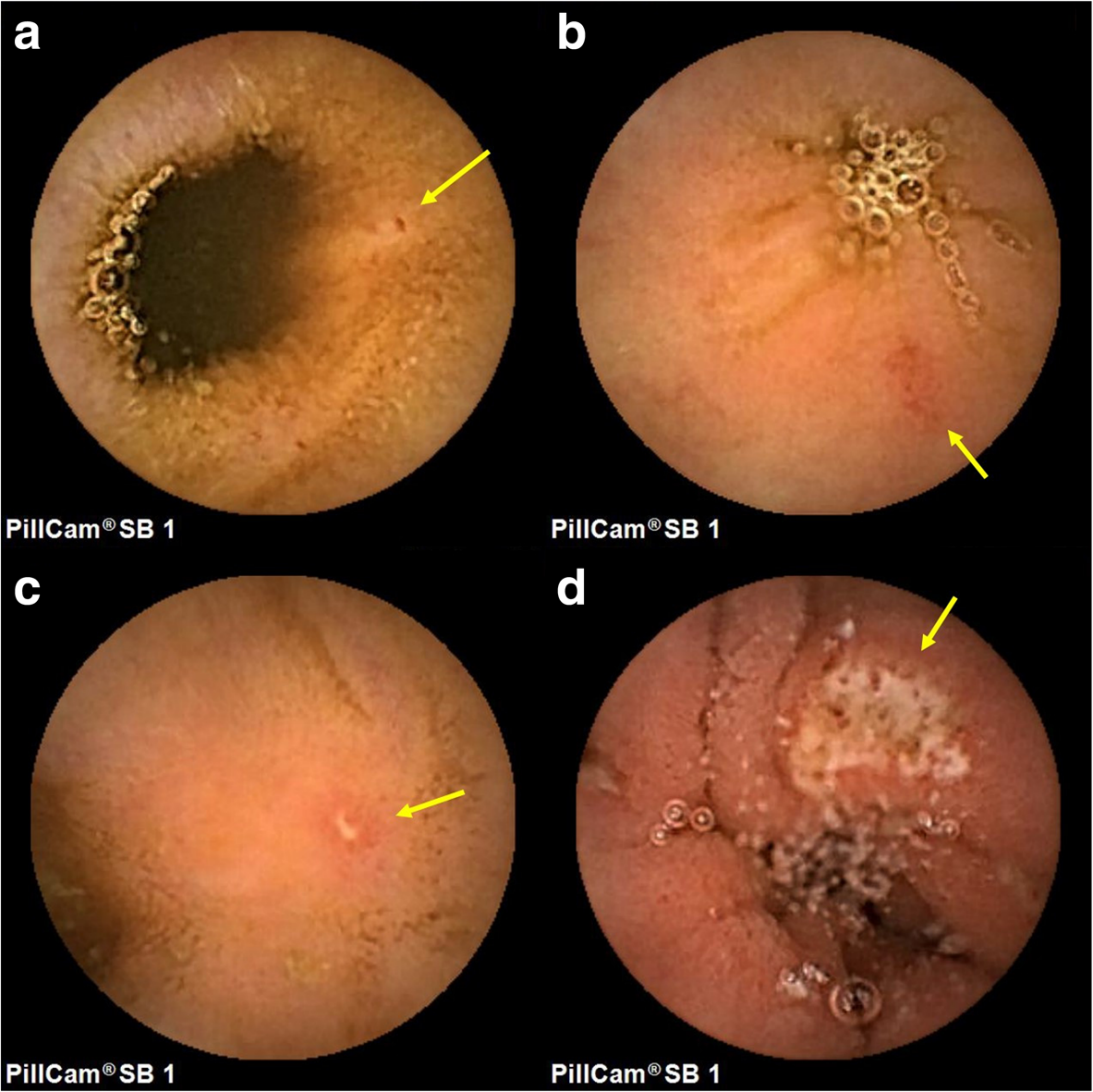
Typical endoscopic findings of a small intestinal mucosal injury: (**a**) spotty redness, (**b**) patchy redness, (**c**) erosion, and (**d**) ulcer

We examined small intestinal lesions using a PillCamSB (Given Imaging, Ltd., Yoquneam, Israel), an SBCE device specifically designed for assessing the small intestine. The SBCE findings were evaluated to identify spotty redness, patchy redness, erosions, and ulcers. Furthermore, if bleeding or stenosis was detected, these findings were appended to those related to mucosal injuries. Spotty redness was defined as a point; patchy redness was defined as red regions with borders extending from the peripheral normal mucosa; erosions were defined as defects in the normal villus mucosa; and ulcers were defined as defects covered with a white coat on the basis of the classifications reported by Fujimori et al. [19] and Niwa et al. [20] with slight modifications (Fig. 1). Evaluation was based on the number of each type of small intestinal mucosal injury.

Two investigators (K.O. and Y.K.) independently assessed the capsule endoscopic images under blinded conditions. If the observers recorded different findings, they discussed the case until consensus was reached.

### Sample size

The sample size was calculated on the basis of the results of a review of the incidence of NSAID-induced small intestinal mucosal injury examined by small intestinal capsule endoscopy. According to several studies, the rate of NSAID-induced small intestinal mucosal injury ranged from 50 to 70% [21–23]. Furthermore, Niwa et al. [20] previously investigated the use of rebamipide for NSAID-induced small intestinal mucosal injury and reported that the incidences of mucosal injury in the placebo and rebamipide groups were 80 and 20%, respectively. We would need to study seven experimental subjects and 14 control subjects to be able to reject the null hypothesis that the failure rates for experimental and control subjects are equal with probability (power) 0.8. The Type I error probability associated with this test of this null hypothesis is 0.05. We used a Pearson’s chi-squared test to evaluate this null hypothesis; therefore, we enrolled 15 subjects in each group.

### Statistical analysis

For continuous or categorical variables, the significance of the differences between groups were determined using the Student’s t test. For paired continuous variables, the significance of the differences between baseline values and those after LDA administration were determined using Wilcoxon’s signed-rank tests. For binary variables, the significance of the differences between groups were determined using Pearson’s chi-squared test. All reported *p*-values were two-sided and values < 0.05 were considered to indicate statistically significant differences. All calculations were performed with JMP® Pro 13 software (SAS Institute Inc., Cary, NC).

## Results

All participants completed the study protocol without complications. Six subjects were excluded from the analysis: one subject had two small intestinal ulcers at baseline, one had three or more small intestinal mucosal injuries at baseline, and four failed to complete two rounds of SBCE examination. A total of 24 subjects were analyzed (group A: 12, group B: 12). There were no significant differences between the two groups with respect to background factors, including age, gender, height, weight, smoking rate, or alcohol consumption rate (Table 1).

**Table 1:** Characteristics of the subjects included in this study

	Group A (*n* = 12)	Group B (*n* = 12)	*p* value
Age, mean ± SD	36.0 ± 6.69	36.8 ± 5.99	0.7749^†^
Gender, male, n (%)	12 (100%)	11 (91.67%)	0.3070^‡^
Height, cm, mean ± SD	172.8 ± 1.73	172.2 ± 1.73	0.7874^†^
Weight, kg, mean ± SD	72.8 ± 2.51	73.0 ± 2.51	0.9630^†^
Smoking history, n (%)	4 (33.3%)	4 (33.3%)	1.000^‡^
Drinking history, n (%)	1 (8.3%)	1 (8.3%)	1.000^‡^

^†^Student’s t-test, ^‡^Pearson’s chi-squared test, SD = standard deviation; LDA = low-dose aspirin

Although there was a significant difference in the median number of small intestinal lesions [range] between baseline and two weeks after LDA administration in group A (baseline: 1 [0–5], after LDA administration: 5 [1–11]; *p* = 0.0059), there was no difference in group B (baseline: 0.5 [0–9], after LDA administration: 3 [0–23]; *p* = 0.0586). In group A especially, the amount of spotty redness significantly increased (baseline: 0 [0–3]; after LDA administration: 2 [0–8], *p* = 0.0039). In group B, although the median number [range] of the lesions at the first tertile of the small intestine did not increase two weeks after LDA administration (baseline: [0–4]; after LDA administration: 1.5 [0–8], *p* = 0.2969), the number of lesions in the second and third tertiles of the small intestine increased significantly (baseline: 0 [0–5]; after LDA administration: 2 [0–15], *p* = 0.0469). In group A, the number of small intestinal lesions increased significantly in each tertile (Tables 2 and 3).

**Table 2:** Comparison of the number of small intestinal lesions between baseline and 2 weeks after LDA administration in group A

		Baseline	After LDA administration	p value
Total small intestine	Spotty redness, median (range)	0 (0–3)	2 (0–8)	0.0039^†^
	Patchy redness, median (range)	1 (0–3)	1 (0–6)	0.2539^†^
	Erosion, median (range)	0 (0–1)	0 (0–4)	0.1250^†^
	All types of lesions, median (range)	1 (0–5)	5 (1–11)	0.0059^†^
The first tertile of the small intestine All types of lesions, median (range)				
		0 (0–2)	2 (0–8)	0.0313^†^
The second and third tertiles of the small intestine All types of lesions, median (range)				
		0 (0–4)	2 (0–6)	0.0156^†^

^†^Wilcoxon signed-rank test. *SD* standard deviation, *LDA* low-dose aspirin

**Table 3:** Comparison of number of small intestinal lesions between baseline and 2 weeks after LDA administration in group B

		Baseline	After LDA administration	p value
Total small intestine	Spotty redness, median (range)	0 (0–6)	1.5 (0–13)	0.1250^†^
	Patchy redness, median (range)	0 (0–3)	1.5 (0–2)	0.3711^†^
	Erosion, median (range)	0 (0–0)	0 (0–9)	0.5000^†^
	All types of lesions, median (range)	0.5 (0–9)	3 (0–23)	0.0586^†^
First tertile of small intestine All types of lesions, median (range)				
		0 (0–4)	1.5 (0–8)	0.2969^†^
Second and third tertiles of the small intestine All types of lesions, median (range)				
		0 (0–5)	2 (0–15)	0.0469^†^

^†^Wilcoxon signed-rank test. SD = standard deviation; LDA = low-dose aspirin

There were no significant differences between the groups in terms of the number of each kind of small intestinal lesion in each tertile, both at baseline and 2 weeks after LDA administration (Tables 4 and 5). Finally, 23 of 24 analyzed patients had some kind of intestinal mucosal injury 2 weeks after administration of LDA; it is believed that comparison of the presence or absence of lesions in chi-squared tests is inappropriate.

**Table 4:** Comparison of the number of small intestinal lesions at baseline between Groups A and B

		Group A	Group B	p value
Total small intestine	Spotty redness, mean ± SD	0.50 ± 1.00	1.0 ± 1.86	0.4206^†^
	Patchy redness, mean ± SD	0.83 ± 0.94	0.75 ± 1.06	0.8398^†^
	Erosion, mean ± SD	0.17 ± 0.39	0.0 ± 0.00	0.1522^†^
	All types of lesions, mean ± SD	1.50 ± 1.62	1.75 ± 2.67	0.7841^†^
First tertile of small intestine All types of lesions, mean ± SD				
		0.58 ± 0.79	0.75 ± 1.29	0.7063^†^
Second and third tertiles of the small intestine All types of lesions, mean ± SD				
		0.92 ± 1.38	1.00 ± 1.54	0.8901^†^

^†^Student’s t test. *SD* standard deviation

**Table 5:** Comparison of the number of small intestinal lesions 2 weeks after LDA administration between groups A and B

		Group A	Group B	*p* value
Total small intestine	Spotty redness, mean ± SD	2.58 ± 2.27	2.75 ± 3.77	0.8968^†^
	Patchy redness, mean ± SD	1.83 ± 2.08	1.17 ± 0.94	0.3227^†^
	Erosion, mean ± SD	0.92 ± 1.38	0.83 ± 2.59	0.9225^†^
	All types of lesions, mean ± SD	5.33 ± 3.60	4.92 ± 6.07	0.8398^†^
First tertile of small intestine All types of lesions, mean ± SD		2.67 ± 2.71	1.92 ± 2.46	0.4856^†^
Second and third tertiles of the small intestine All types of lesions, mean ± SD		2.67 ± 1.92	2.83 ± 4.02	0.8981^†^

^†^Student’s t test. *SD* = standard deviation

## Discussion

Although some clinical studies have reported that gastric mucoprotective drugs might prevent or cure LDA-induced small intestinal mucosal injury [2, 6–10], no reports have assessed the effectiveness of ES. In our previous experimental study on rats, we determined that ES did not prevent indomethacin-induced small intestinal mucosal injury [14]. However, this previous study was limited, because the dose of ES may have been too low for the prevention of small intestinal mucosal injury. Therefore, we determined the efficacy of a double dose of ES for the prevention of LDA-induced small intestinal mucosal injury. In this study, we demonstrated that a double dose of ES prevented LDA-induced small intestinal mucosal injury in healthy volunteers. Because this was a short-term study (2 weeks), it was difficult to identify any differences in LDA-induced small intestinal erosions or ulcers; however, small mucosal changes, such as spotty redness, could be examined well.

It is believed that ES does not have an effect on the lower portion of the small intestine because its effects have already been exhausted in the LDA-induced mucosal injuries in the stomach or the upper portion of the small intestine. Therefore, when gastric mucosal injury is prevented by concomitant use of PPI in advance, ES might have an effect on small intestinal mucosal injuries, because the drug is not absorbed in the stomach. Encapsulated ES might be one option for drug delivery to the lower portion of the small intestine. Furthermore, high-dose ES might be also an effective method for drug delivery.

Mechanisms underlying the effect of ES on the stomach include anti-bacterial activity, enhanced mucosal protection, and anti-peptic-activity [24–26]. The mechanisms underlying the effects on the small intestine are unclear. Recently, in a study using a zebrafish experimental animal model, ES alleviated neomycin-induced hair cell damage through reactive oxygen species scavenging or anti-apoptotic effects [27]. ES might prevent LDA-induced small intestinal mucosal injury through similar mechanisms.

This study had several limitations. First, it was conducted in healthy volunteers for a short period. In our previous report, there was a likelihood that the severity of LDA-induced small intestinal mucosal injury differed between healthy volunteers and real LDA users. For the real LDA users, the severity of LDA-induced small intestinal mucosal injury was related to the periods of LDA administration [1]. Second, we did not evaluate mucosal lesions in the stomach. ES might have already been exhausted in the stomach owing to gastric mucosal injuries.

## Conclusions

We demonstrated that ES prevented LDA-induced small intestinal mucosal injury. However, this effect was noted in the upper part of the small intestine only. To clarify the true efficacy of ES for preventing LDA-induced small intestinal mucosal injury, studies of actual LDA users are required.

## Abbreviations

ES: Ecabet sodium
LDA: Low-dose aspirin
NSAIDs: Non-steroidal anti-inflammatory drugs
PPI: Proton pump inhibitor
SBCE: Small bowel capsule endoscopy
